# A Balanced Approach to Adaptive Probability Density Estimation

**DOI:** 10.3389/fmolb.2017.00025

**Published:** 2017-04-25

**Authors:** Julio A. Kovacs, Cailee Helmick, Willy Wriggers

**Affiliations:** Department of Mechanical and Aerospace Engineering, Old Dominion UniversityNorfolk, VA, USA

**Keywords:** adaptive density estimation, covariance ellipsoid, covariance smoothing, optimal number of nearest neighbors, R^*^-tree, visual criterion

## Abstract

Our development of a Fast (Mutual) Information Matching (FIM) of molecular dynamics time series data led us to the general problem of how to accurately estimate the probability density function of a random variable, especially in cases of very uneven samples. Here, we propose a novel Balanced Adaptive Density Estimation (BADE) method that effectively optimizes the amount of smoothing at each point. To do this, BADE relies on an efficient nearest-neighbor search which results in good scaling for large data sizes. Our tests on simulated data show that BADE exhibits equal or better accuracy than existing methods, and visual tests on univariate and bivariate experimental data show that the results are also aesthetically pleasing. This is due in part to the use of a visual criterion for setting the smoothing level of the density estimate. Our results suggest that BADE offers an attractive new take on the fundamental density estimation problem in statistics. We have applied it on molecular dynamics simulations of membrane pore formation. We also expect BADE to be generally useful for low-dimensional applications in other statistical application domains such as bioinformatics, signal processing and econometrics.

## 1. Introduction

One of the most popular non-parametric density estimation methods is *kernel density estimation* (KDE), whereby the density is estimated by means of a sum of kernel functions centered at the sample points (Silverman, 1986; Wand and Jones, 1995):

(1) $$\hat{f}(x)=\frac{1}{M}\sum_{j\;=\;1}^{M}K_{H}(x-x_{j}),$$

where $K_{H}\left(\text{x}\right)=\det{\left(H\right)}^{-1/2}K\left(H^{-1/2}\cdot \text{x}\right)$, *K* : ℝ^*d*^ → ℝ being the *d*-variate kernel and *M* the data size. One of the most commonly used kernels is the Gaussian: $K\left(\text{x}\right)=C_{d}\exp\left(-∥\text{x}{∥}^{2}/2\right)$, with *C*_*d*_ a normalizing constant that depends on the dimension *d*. The *d*×*d* matrix *H*, called the *bandwidth matrix*, could either be fixed, or it could depend on the sample point **x**_*j*_ (“sample point estimator”) or on the test point **x** (“balloon-type estimator”).

Originally, we adopted a fixed-bandwidth KDE approach in our recent application to Fast (Mutual) Information Matching (FIM) of molecular dynamics time series data (Kovacs and Wriggers, 2016). The fixed-bandwidth approach is well mature and there exist a wide range of methods for bandwidth selection (see e.g., Jones et al., 1996 for a survey). Among these, the method by Sheather and Jones (1991) could be regarded as the *de facto* standard in the univariate case (Jones et al., 1996). Our application to molecular dynamics time series relies on non-negative activity functions (Kovacs and Wriggers, 2016). As discussed in more detail by Wriggers et al. (2017), the graph-based activity functions we typically use are zero during quiescent time periods of the simulation, leading to an uneven distribution of activity values with a strong peak at zero that is not amenable to fixed-bandwidth KDE approaches. In protein simulations we have therefore recommended to use a rms-fluctuation-based activity that gives a more even histogram (Kovacs and Wriggers, 2016). Unfortunately this is not an option for the membrane simulations in the accompanying paper (Wriggers et al., 2017), so we require a variable-bandwidth approach that can handle graph-based activity functions in that application.

The situation in regard to the variable-bandwidth KDE methods is less well developed. In fact, it has not been easy to make significant performance improvements by allowing the bandwidth to vary from point to point (Farmen and Marron, 1999). Several approaches have been proposed, with varying degrees of success across different types of data sets. One of the earliest approaches was that of Breiman, Meisel and Purcell, who used bandwidths proportional to the distance from each sample point to its *k*th nearest neighbor (Breiman et al., 1977). So, for dimensions *d* > 1 the *j*-dependent bandwidth matrices are scalar (i.e., multiples of the identity matrix). Later, Abramson (1982) proposed a square-root law, whereby the bandwidth at each point is taken to be inversely proportional to the square root of the density. Since the actual density is not known, a “pilot density” is needed, which is usually computed using a fixed-bandwidth method. Like the previous approach, in *d* > 1 it produces bandwidth matrices that are scalar.

One of the earliest alternative approaches to improve the performance of variable bandwidth estimators was proposed by Sain and Scott (1996): the binned kernel estimator, in which the support of the density is divided in *m* equal parts. Each of these subintervals yields a value of the bandwidth, which is then used for the kernels centered at points belonging to the corresponding subinterval. This method was extended to the multivariate setting by Sain (2002). Hazelton (2003) refined this approach (in the univariate case) by using cubic splines instead of piecewise-constant functions to model the bandwidths, showing improvements in the quality of the density estimates. However, these approaches are very slow, as they involve an optimization problem over many variables. Brewer (2000) showed improved results relative to Sain and Scott (1996) by using a Bayesian approach based on likelihood cross-validation, which works specially well for small sample sizes, and adds a local smoothing step to enhance the visual appeal of the density estimates. This method was extended by Zougab et al. (2014) to the multivariate case, in which the bandwidth matrices are not restricted to being diagonal. Like Brewer's approach, it works very well for small sample sizes, but the complexity scales quadratically with the sample size.

Attempts at alleviating the mentioned limitations include a class of methods that use convex combinations (i.e., linear combinations with non-negative coefficients adding up to 1) or mixtures of densities of certain types. Vapnik and Mukherjee (2000) used a mixture of Gaussian densities in which the coefficients are optimized by matching the sample's cumulative distribution function (CDF) with the CDF estimator. The Gaussian densities are isotropic (i.e., having scalar covariance matrices). Song et al. (2008) assume the density to be a convex combination of several prototype densities, and optimizes the coefficients by matching the mean estimators. The prototype densities are Gaussians with diagonal covariance matrices. Ganti and Gray (2011) proposed a density estimator in which the kernel functions are convex combinations of isotropic Gaussians of various widths. The expected outcome is that this would produce a richer set of function shapes which would compensate the limitation arising from using isotropic Gaussians. However, the quality of the resulting density estimates (judged by visual inspection) is questionable.

Several other interesting ideas have also been put forward. For instance, Katkovnik and Shmulevich (2002) described a univariate balloon-type estimator based on the “intersection of confidence intervals” (ICI) rule (i.e., shrinking sequences of intervals), for which, at each test point *x*, a fixed, arbitrary sequence of increasing bandwidth values is scanned until the ICI criterion is met, yielding the bandwidth for that point. Wu et al. (2007) used a cluster analysis of the set of nearest neighbors to derive the bandwidths at each sample point. The analysis is restricted to isotropic (scalar) bandwidth matrices. Shimazaki and Shinomoto (2010) used a “local MISE” criterion, which includes a window factor in the integral defining the mean squared integrated error (MISE) to derive local bandwidths in the univariate case. The “Rodeo” approach (Liu et al., 2007) is specially suited for high-dimensional data. The density is assumed to be the product of a non-parametric factor and a parametric one, which is known either completely or up to finitely many parameters. Bandwidth matrices are restricted to being diagonal, and a sparsity condition has to be imposed for the problem to be tractable.

Motivated by the various limitations of previous methods, here we propose a novel approach, which we call “BADE” (for Balanced Adaptive Density Estimation) that offers several desirable features: good scaling for large data sizes (sublinear complexity in *M* for *d* = 1and2); not restricted to diagonal bandwidth matrices; free of data-dependent parameters (the user does not need to make any choices). In fact, we are no longer dealing with bandwidth matrices *per se*, although there is a connection with kernel estimation through the “effective” number of neighbors (Section 2.1).

## 2. Balanced adaptive density estimation

Let $\text{P}=\left\{{\text{p}}_{1},\ldots ,{\text{p}}_{M}\right\}\subset {\mathbb{R}}^{d}$ be a sample of size *M* drawn independently from an unknown *d*-dimensional distribution having probability density function *f* : ℝ^*d*^ → ℝ. Let Σ_**P**_ be the covariance matrix of **P**, which we use as an estimate of the covariance matrix of the true distribution. This matrix will be used later to give us an idea of the global size and shape of the whole sample.

Unlike most of the previous approaches, we do not use a kernel-based estimation approach. Instead, the basic idea is the following: for each probe point **x** ∈ ℝ^*d*^ where we want to estimate the density, we determine the set *N*_*k*_(**x**) of its *k* nearest neighbors among **P**, and compute its covariance matrix:

(2) $$\Sigma_{k}(x)=\mathrm{Cov}(N_{k}(x)),$$

which gives us a basic description of the size and shape of the set of sample points near **x**, by means of the “covariance ellipsoid” (or “inertia ellipsoid”) defined by the eigenvectors and eigenvalues of this matrix. The volume (modulo a constant) of that ellipsoid is $V_{k}\left(\text{x}\right)=\sqrt{\det\Sigma_{k}\left(\text{x}\right)}$. (Recall that the determinant is the product of the eigenvalues, which are the squares of the corresponding principal axes of the ellipsoid.) Then, our first version for the density estimate at **x** is:

(3) $$\hat{f}(x)=C\cdot \frac{k}{MV_{k}(x)},$$

where *C* is a scaling constant. In practice, the density estimate is computed, omitting *C*, on a grid covering the sample **P**, and then *C* is determined so that the integral of $\hat{f}$ is 1.

Of course, this expression is very reminiscent of the original proposal of Loftsgaarden and Quesenberry (1965), just with the volume of the ellipsoid in place of the volume of the sphere of radius equal to the distance from **x** to the *k*th nearest sample point. It is well known that Loftsgaarden and Quesenberry's method produce heavy tails and spiky density estimates (Silverman, 1986). The spikiness is due to the use of the simple *k*th nearest neighbor, which is highly variable. The use of *V*_*k*_(**x**) drastically decreases this variability, since this ellipsoid—being the covariance ellipsoid of a set of *k* neighbors— is much more stable than a domain (whether spherical or ellipsoidal) whose size is based simply on the distance to the *k*th neighbor. Note that this ellipsoid does not, in general, contain the set of neighbors on which it is based.

### 2.1. Fixing heavy tails

The basic idea described above still suffers from a number of drawbacks. First, as with the method of Loftsgaarden and Quesenberry (1965), this basic idea produces heavy tails—since the set of nearest neighbors remains virtually constant as the point **x** moves away (and exactly constant in the univariate case). This can be remedied by introducing a decay factor, giving an “effective” *k*:

(4) $$k_{e}(x)=k\cdot \exp[-\frac{1}{2}(x-\mu_{k}(x))\cdot \Sigma_{k}{\left(x\right)}^{-1}\cdot {\left(x-\mu_{k}(x)\right)}^{T}],$$

where μ_*k*_(**x**) = *Mean*(*N*_*k*_(**x**)). This factor follows a decay rate corresponding to the distribution of the *k* nearest neighbors of **x**, and is useful in the “interior” of the set **P** as well as the “exterior.” Thus, our second version for the density estimate is

(5) $$\hat{f}(x)=C\cdot \frac{k_{e}(x)}{MV_{k}(x)}.$$

We note that due to the exponential decay of *k*_*e*_(**x**), this estimator is integrable. Incidentally, we can write Equation (5) as follows (using the kernel notation as in Equation 1):

(6) $$\hat{f}(x)=\frac{C}{M}\cdot k\cdot K_{\Sigma_{k}(x)}(x-\mu_{k}(x))\approx \frac{C}{M}\cdot \sum_{l\;=\;1}^{k}K_{\Sigma_{k}(x)}(x-p_{r_{l}}),$$

where {**p**_*r*_*l*__ | 1 ≤ *l* ≤ *k*} = *N*_*k*_(**x**). Thus, the estimator given by Equation (5) is approximately like a balloon-type Gaussian kernel estimator, but based only on the *k* nearest neighbors of the probe point **x**, instead of all the sample points.

### 2.2. Determination of *k*

A second drawback of our basic idea is: what should *k* be? Loftsgaarden and Quesenberry (1965) take it as independent of **x**, depending only on the sample size *M*. We can improve on this by choosing *k* in such a way that the volume *V*_*k*_(**x**) of the covariance ellipsoid be a certain function of *f*(**x**). Two common choices, in a sense antipodal to each other, are:

1. Volume = const. This would yield a *k* that is approximately proportional to the density.
2. Volume = const/*f*. This would yield a *k* that is approximately constant.

We found that neither of these extremes produces good density estimates: a constant volume is essentially like a histogram: it will not resolve sharp enough peaks, and will yield zero in regions where the sample points are widely spread; a constant *k* will tend to be too large in region of low density, and too small in regions of high density.

However, the geometric mean of both offers a good compromise: $Volume=const/\sqrt{f}$. (This is why we named our approach “balanced.”) Hence, we have the equation

(7) $$V_{k}(x)=\text{const}/\sqrt{f(x)}.$$

**Figure 1:**
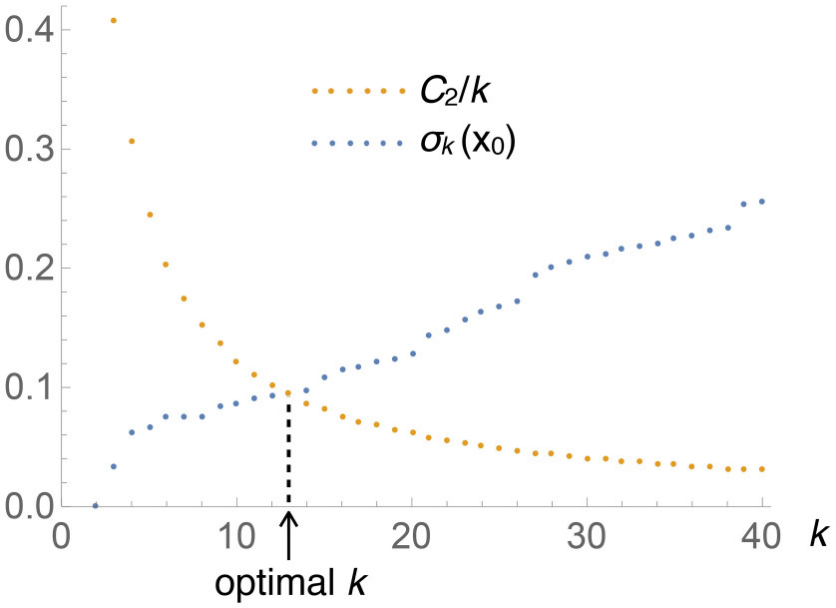
**Graphical illustration of the determination of the optimal number of nearest neighbors, *k*, to be used at each test point *x* (this particular plot corresponds to the univariate Old Faithfual data set, at *x*_0_ = 3.59)**. The intersection of both curves provides the solution to Equation (8).

For *f*(**x**) we can use, in this equation, the estimate *f*(**x**) ≈ *C*_1_*k*/*V*_*k*_(**x**). This allows us to solve the equation for *V*_*k*_(**x**):

(8) $$V_{k}(x)=\frac{C_{2}}{k},$$

where *C*_2_ (which depends on *M* but not on **x**) subsumes the various constants. Figure 1 depicts the situation graphically: when *k* is small, the left-hand side of the equation (i.e., *V*_*k*_(**x**)) is small, while the right-hand side (*C*_2_/*k*) is large, and vice versa. The point where the two curves cross gives the optimal *k* for this **x**. Solving this equation is easy: keep increasing *k* by 1 until the inequality

(9) $$V_{k}(x)\cdot k<C_{2}$$

no longer holds. This can be efficiently implemented in code by means of incremental nearest neighbor methods (Hjaltason and Samet, 1999), in which the cost of retrieving each additional neighbor is essentially *O*(1) (see “Complexity,” below).

### 2.3. Determination of *C*_2_

The constant *C*_2_ in Equation (8) depends on *M*, *d*, and *f*. As pointed out in the introduction, one of our goals was to devise a method that does not depend on parameters that have to be adjusted for each particular data set. Our method satisfies this condition (as we'll describe in a moment) except for the dependence on the dimension *d*, which has to be worked out for each *d*. (We worked out the values for *d* = 1 and 2 since these are the ones that most interest us for our applications).

In *d* > 1 the data needs to be rescaled so that each coordinate have unit variance. This is important not only for the derivation of the expression of the constants, but also for the correct functioning of the nearest neighbor search: if the rescaling were not done, then the neighbor search would be as if using ellipsoids instead of spheres for its distance queries. (We preferred this transformation rather than sphering—making the covariance matrix the identity—since the latter changes the correlations, being a skewed transformation.) In these conditions, we factor the volume of the covariance ellipsoid of the whole sample (square root expression in this equation) out of *C*_2_:

(10) $$C_{2}=H_{0}\sqrt{\det\Sigma_{P}}.$$

It turns out that *H*_0_ does not depend on *f*, but only on *M* and *d*. We verified this by means of a “visual criterion.” The reason we chose this type of criterion, instead of a more objective one such as MISE, is that good MISE performance does not guarantee visually appealing density estimates (Farmen and Marron, 1999), which is one of our goals. One such visual criterion was proposed by Marron and Tsybakov (1995), in which the distance between the graphs of both functions is evaluated, instead of the vertical distance. Here we needed a different type of criterion to determine *H*_0_: we ourselves examined by eye the density estimates resulting from an array of values of *M* and *H*_0_, for various simulated densities. For each *M* and density, we looked for the minimum value of *H*_0_ that yielded a density estimate that did not look undersmoothed. Even though this visual criterion might seem rather *ad hoc*, it actually yielded surprisingly good linear relationships in log/log scale on the *H*_0_/*M* plane. The fitted lines, which were independent of the particular density, correspond to the following power laws:

(11) $$H_{0}=\{\begin{array}{ll} 0.028\;M^{4/5} & \text{for }d\;=\;1,\\ 0.162\;M^{2/5} & \text{for }d\;=\;2. \end{array}$$

It is interesting to note that the expression for *d* = 2 is nearly equal to the square root of that for *d* = 1: $\sqrt{0.028M^{4/5}}=0.167M^{2/5}$. This is reassuring and adds confidence to our visual criterion, in addition to providing an obvious conjecture about the expression for *H*_0_ for *d* > 2 (which we haven't tested).

A more theoretical justification of the expression for *H*_0_ would probably be related to how the human visual system processes information. One possible approach could be the addition of a regularization term that would emulate visual perception. An intriguing link to the standard MISE theory in kernel density estimation is that the optimal bandwidth, in the 1-dimensional case, is proportional to *M*^−1/5^, which is *H*_0_/*M* (Wand and Jones, 1995).

We emphasize that this visual criterion was used only as a premise to determine the optimal dependence (on *M*) of the coefficient *C*_2_. This optimal dependence is determined once and for all—the user does not need make any choices. However, the user could, with discretion, vary the coefficients in the formula for *H*_0_ (Equation 11), to obtain density estimates with greater or lesser amount of smoothing than that provided by the values in Equation (11). As a rule of thumb, our visual tests (not shown) suggest to keep the variation within a factor 2 from the stated values.

### 2.4. Covariance smoothing

To further improve the visual appeal of the density estimate given by Equation (5), we added an optional smoothing step to our method. The smoothing procedure was inspired by that of Brewer (2000), who averages the inverse variances of two neighboring sample points on either side of each sample point, producing estimates that are relatively free from unnecessary minor fluctuations. Since our approach is grid-based, we need a more sophisticated procedure, as averaging inverse variances of neighboring grid points would not be correct, since the spacing is arbitrary. We need to weight the contributions of all the grid points according to their respective covariance matrices and locations relative to the test point. Denoting the grid points by **x**_*j*_ (*j* = 1, …, *G*), where *G* is the size of the grid, and the corresponding covariance matrices (Equation 2) by Σ(**x**_*j*_) (omitting for clarity the subindex that indicated the number of nearest neighbors used), we define the smoothed precision matrices by:

(12) $${\hat{\Sigma }}_{i}^{-1}=\frac{\sum_{j\;=\;1}^{G}w_{i,j}\Sigma {\left(x_{j}\right)}^{-1}}{\sum_{j\;=\;1}^{G}w_{i,j}},$$

where the weights (influence of point *j* on point *i*) are given by

(13) $$w_{i,j}=\frac{1}{\sqrt{\det\Sigma (x_{j})}}\cdot \exp[-\frac{1}{2}(x_{i}-x_{j})\cdot \Sigma {\left(x_{j}\right)}^{-1}\cdot {\left(x_{i}-x_{j}\right)}^{T}].$$

Thus, the contribution of each covariance matrix is in accordance with the value of the Gaussian function defined by it, at each of the grid points. This equation shows that the smoothing can be considered local, in the sense that points **x**_*j*_ where Σ(**x**_*j*_) is large (where the density is low) or which are far from **x**_*i*_ contribute little, and only points that are close to **x**_*i*_ and with a small Σ(**x**_*j*_) will contribute significantly to ${\hat{\Sigma }}_{i}$. (Note: “large” or “small” applied to a matrix mean that the volume of its ellipsoid—or equivalently, its determinant, or the product of its eigenvalues— is large or small).

Since both the smoothing step just described and the main step (Equation 5) are local, we see that our method does not suffer from the non-locality issues that affect, for instance, one version of Abramson's square-root method (basically, extreme tail sample points affect the density estimate elsewhere too much; see Terrell and Scott, 1992; Hall et al., 1995 for details).

Finally, we also need the smoothed version of the “effective” *k* values (Equation 4). They are computed similarly to Equation (12):

(14) $${\hat{k}}_{e,i}=\frac{\sum_{j\;=\;1}^{G}w_{i,j}k_{e,j}}{\sum_{j\;=\;1}^{G}w_{i,j}}.$$

Then, the smoothed version of the density estimate is given by

(15) $$\hat{f}(x_{i})=C\cdot \frac{{\hat{k}}_{e,i}}{\sqrt{\det{\hat{\Sigma }}_{i}}},$$

where the constant *C* is determined by the condition of $\hat{f}$ integrating to 1.

### 2.5. Complexity

We analyze separately the two steps of our method: the main estimator (Equation 5) and the (optional) covariance-smoothing step (Section 2.4).

The first, main step requires the incremental retrieval, for each test point **x**, of successive nearest neighbors. In two and higher dimensions, the R^*^-tree data structure (Beckmann et al., 1990) provides an effective means to implement such a retrieval procedure (Hjaltason and Samet, 1999). This procedure makes use of “priority queues” or heaps, one of its most efficient implementations being the *pairing heap* (Fredman et al., 1986), in which the cost of an insertion operation is *O*(1). Using this implementation, the cost of finding *k* nearest neighbors among *M* data points in 2 dimensions turns out to be *O*(*k*log*M*) (Hjaltason and Samet, 1999). (The complexity analysis gets more complicated in higher dimensions; see Hjaltason and Samet (1999) for details. In dimension 1, determining the sequence of nearest neighbors is a simple logarithmic-time procedure which does not require the use of any special data structure.) The number *k* will vary from point to point, but always *k* ≤ *M*, and so the per-point cost would be ≤ *O*(*M*log*M*). However, this is not a typical situation, as the average *k* will usually be much less than *M*. If fact, more realistic estimates for the average *k* are of the order *O*(*M*^1/2^) (Loftsgaarden and Quesenberry, 1965; Silverman, 1986). Hence, the total cost of the main step can be approximated by

(16) $$T_{1}=\{\begin{array}{ll} O(G\log\;M) & \text{for }d\;=\;1,\\ O(GM^{1/2}\log\;M) & \text{for }d\;=\;2. \end{array}$$

where *G* is the size of the grid. We note that an incremental implementation of the nearest-neighbor search is essential to achieve this low complexity. Algorithms that are not incremental need to recompute the whole set of nearest neighbors each time one more is needed, with a significant deterioration in the efficiency.

**Figure 2:**
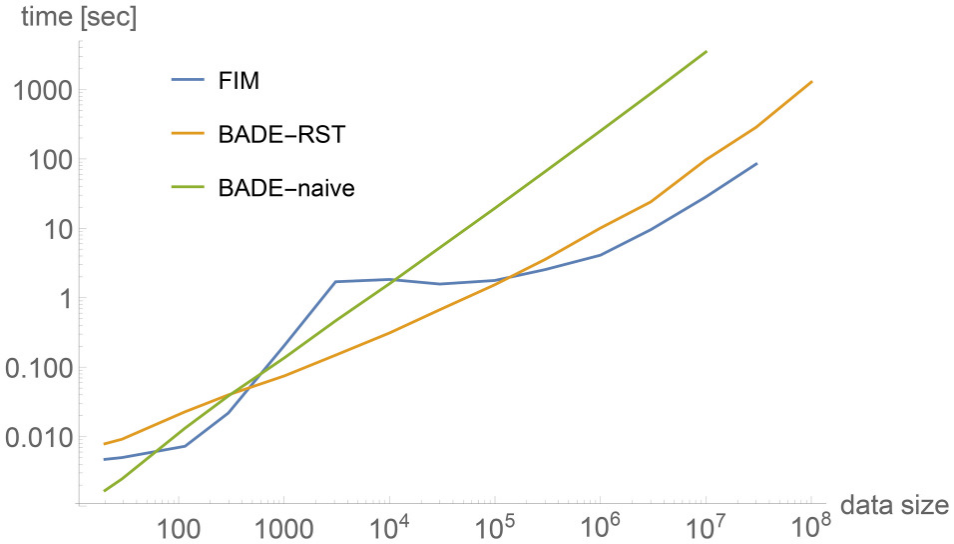
**Comparison between timings obtained by three methods applied to 2-dimensional data sets**. The data sets were artificially generated to simulate a bimodal distribution, shown in Figure 3A for *M* = 1, 000. See the section on Complexity for more details.

**Figure 3:**
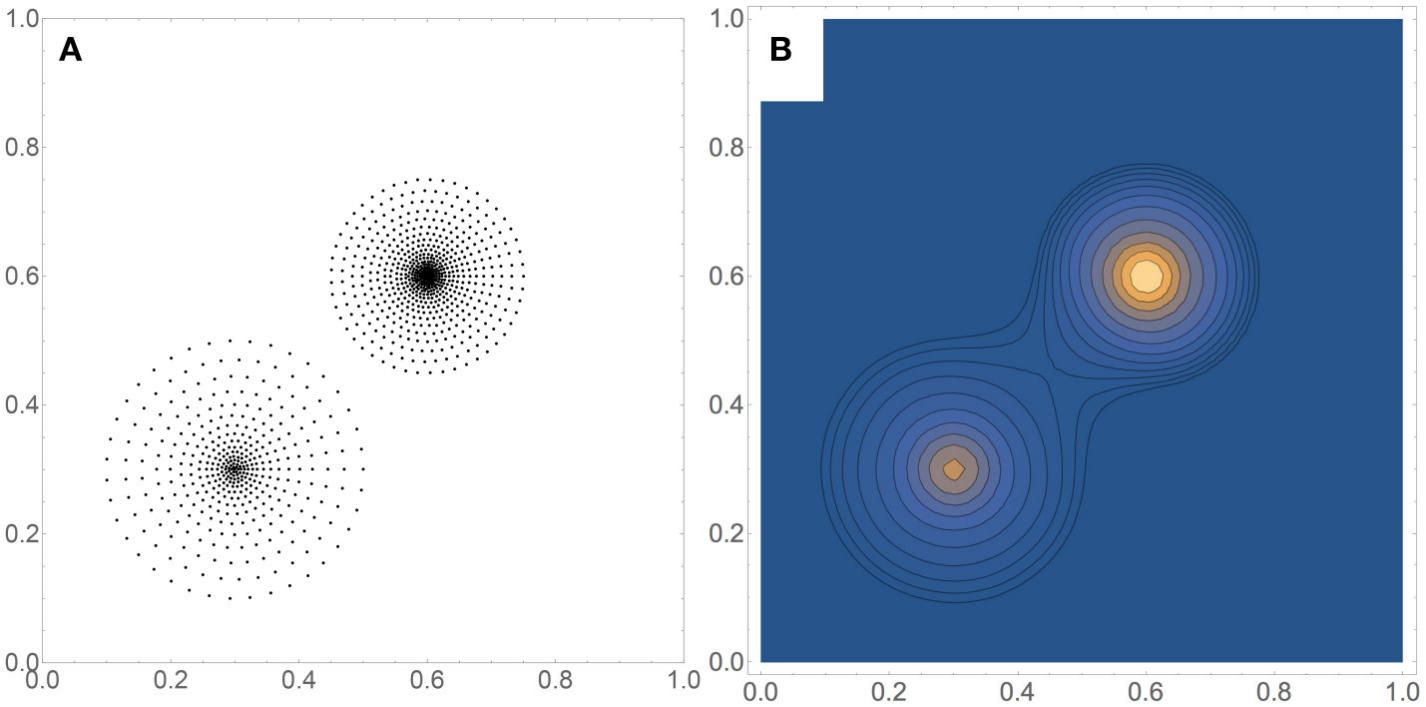
**(A)** Example of a data set used for the timing comparison shown in Figure 2. Here *M* = 1, 000. **(B)** Corresponding contour plot of the density estimate, which includes the covariance-smoothing step.

Figure 2 shows a comparison between timings obtained by three methods applied to 2-dimensional data sets of a wide range of sizes, from *M* = 20 to 10^8^. The data sets were artificially generated to simulate a bimodal distribution, shown in Figure 3A for *M* = 1, 000, with the corresponding density estimate shown in Figure 3B. The three methods were: (a) FIM (using a fixed bandwidth) (Kovacs and Wriggers, 2016); (b) BADE-RST using the R^*^-tree to retrieve nearest neighbors; (c) BADE-naive using a naive way to retrieve nearest neighbors (i.e., by sorting the data points according to their distances to each probe point). We can see that FIM and BADE-RST have very similar asymptotics. In fact, FIM has a complexity of *O*(*M*) (Kovacs and Wriggers, 2016), which is slightly worse than that of BADE-RST, although the constant is smaller for FIM. However, FIM only computes the mutual information, not the whole density function as BADE does, which introduces the factor *G* in Equation (16).

As for the second step (covariance smoothing), Equations (12) and (14) tell us that the cost will be

(17) $$T_{2}=O(G^{2}),$$

where the constant can be made quite small by summing each Gaussian function only over the ellipsoid where it has significant values (usually a small fraction of the total volume).

## 3. Results

**Figure 4:**
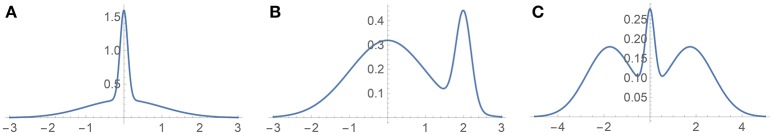
**Test densities (Gaussian mixtures) used in our univariate simulation study**. **(A)** H3 (Kurtotic unimodal); **(B)** H4 (Skewed bimodal); **(C)** H5 (Trimodal).

**Figure 5:**
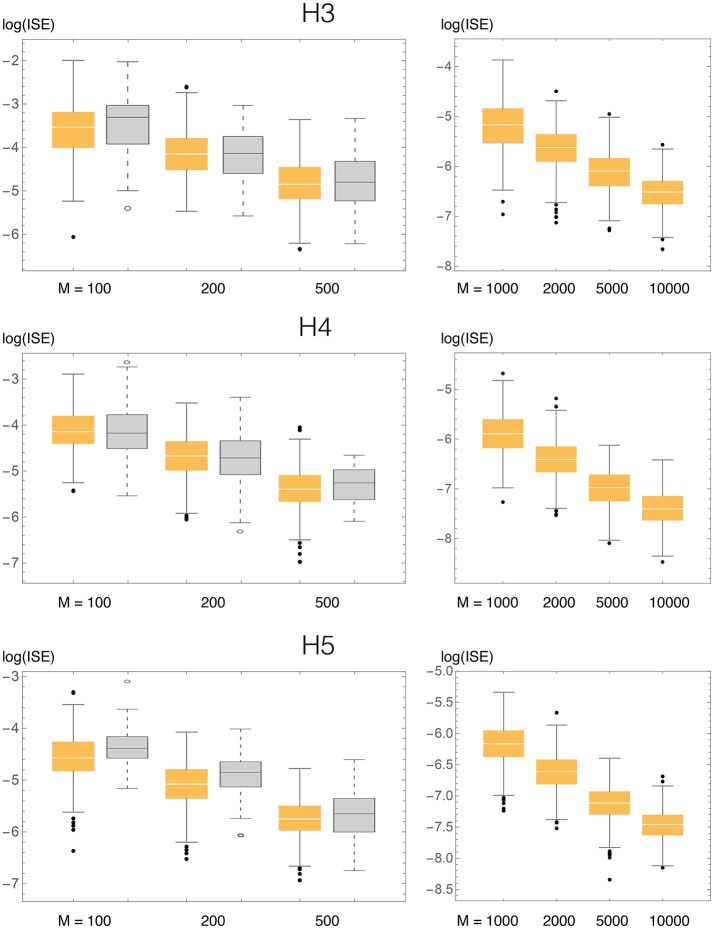
**ISE statistics for the three univariate test densities (H3, H4, H5), compaing our method (BADE, in orange) and VB3 (Hazelton, 2003, in gray)**. Also shown are results for larger values of the sample size *M*, not considered by Hazelton.

In order to evaluate the accuracy of BADE, we performed statistics of the ISE (Integrated Squared Error) for simulated samples taken from known distributions (Figures 4, 5 for the univariate case; **Figures 9**, **10** for the bivariate case). The ISE of an estimator $\hat{f}$ is defined as

(18) $$\text{ISE}=\int (\hat{f}(x)-f(x){)}^{2}dx\approx \Delta x_{1}\cdots \Delta x_{d}\cdot {\sum_{j\;=\;1}^{G}\left(\hat{f}(x_{j})-f(x_{j})\right)}^{2}.$$

**Figure 6:**
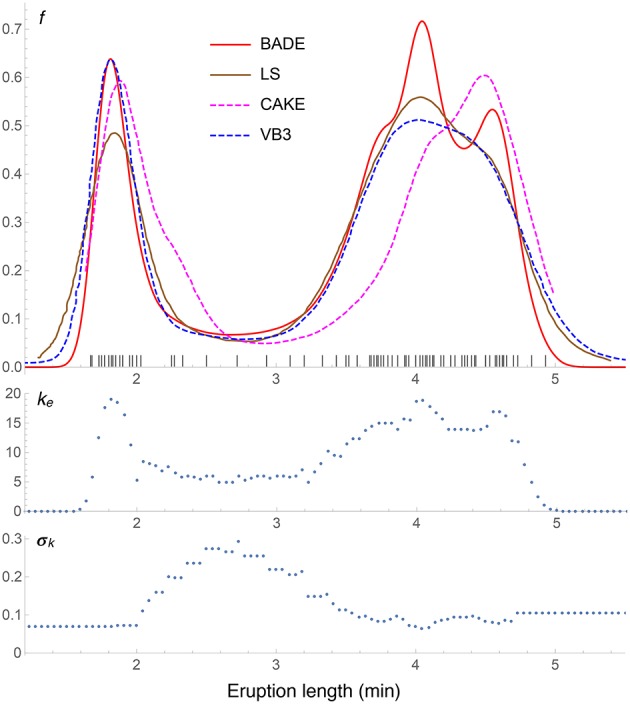
**Density estimates for the univariate Old Faithful data set, comparing our method (BADE) with LS (Brewer, 2000), CAKE (Ganti and Gray, 2011), and VB3 (Hazelton, 2003)**. The second and third rows show, respectively, the optimal effective number of nearest neighbors, and the standard deviation of the set of the *k* nearest neighbors, at each point of a regular grid of size 100. The marks on the *x* axis indicate the sample points.

**Figure 7:**
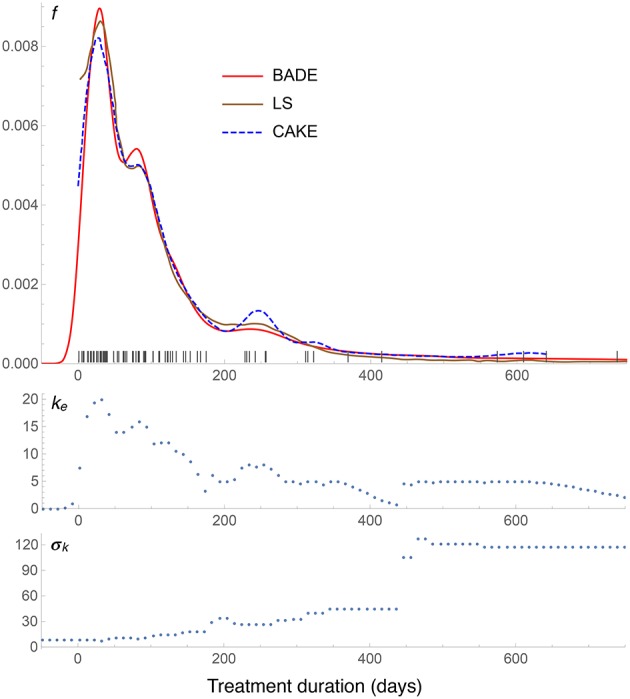
**Density estimates for the suicide data set, comparing our method (BADE) with LS (Brewer, 2000) and CAKE (Ganti and Gray, 2011)**. The second and third rows show, respectively, the optimal effective number of nearest neighbors, and the standard deviation of the set of the *k* nearest neighbors, at each point of a regular grid of size 100. The marks on the *x* axis indicate the sample points.

**Figure 8:**
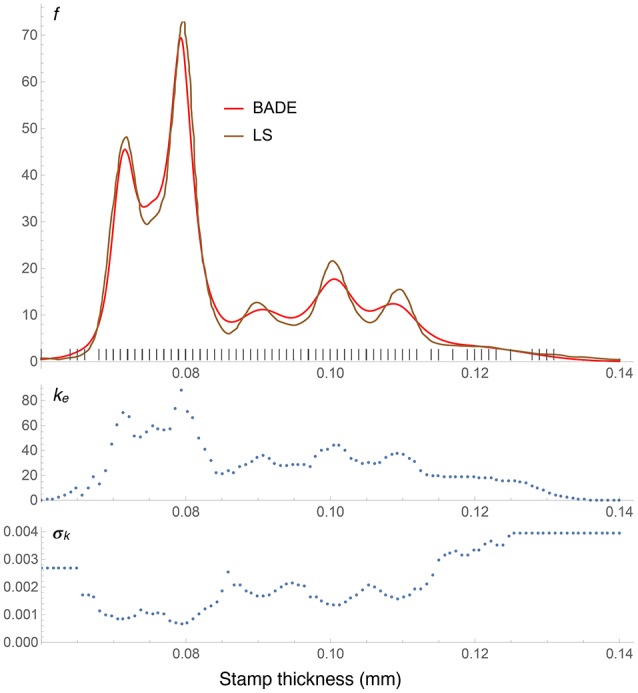
**Density estimates for the Hidalgo stamp data set, comparing our method (BADE) with LS (Brewer, 2000)**. The second and third rows show, respectively, the optimal effective number of nearest neighbors, and the standard deviation of the set of the *k* nearest neighbors, at each point of a regular grid of size 100. The marks on the *x* axis indicate the sample points; notice the equispacing due to rounding.

Also, we considered some real data sets to compare the density estimates of BADE with those of previous methods (Figures 6–8 for the univariate case; **Figures 11**, **12** for the bivariate case). The BADE results were computed using Equation (15), i.e., including the covariance-smoothing step.

### 3.1. Univariate case

#### 3.1.1. Simulated examples

The three univariate simulated densities, all Gaussian mixtures, on which we tested our method are shown in Figure 4. They are densities 3, 4, and 5 used by Hazelton (2003), so we will refer to them in this paper as H3, H4, and H5:

1. **H3** (Kurtotic unimodal, equal to density #4 in Marron and Wand, 1992): $\frac{2}{3}\text{N}\left(0,1\right)+\frac{1}{3}\text{N}\left(0,\frac{1}{10}\right)$. (We denote the normal distribution with mean μ and standard deviation σ by N(μ, σ)).
2. **H4** (Asymmetric bimodal, similar to density #8 in Marron and Wand, 1992): $\frac{4}{5}\text{N}\left(0,1\right)+\frac{1}{5}\text{N}\left(2,\frac{1}{5}\right)$.
3. **H5** (Symmetric trimodal, similar to density #9 in Marron and Wand, 1992): $\frac{9}{20}\text{N}\left(-\frac{7}{4},1\right)+\frac{9}{20}\text{N}\left(\frac{7}{4},1\right)+\frac{1}{10}\text{N}\left(0,\frac{1}{5}\right)$. (Note the typo in Table 1 of Hazelton's paper in the equation for this density).

We compared the ISE statistics of our method, for each of the above three densities, with those of Hazelton (2003). They are displayed, in logarithmic scale, in Figure 5. We also considered larger sample sizes *M*, up to 10,000. For each density and sample size, 500 simulated samples were produced. We can observe that in most cases the ISE values of our method (BADE) are lower that those of Hazelton's method (VB3). The exceptions are H4 with *M* = 100 and 200, for which they are virtually the same. In some cases we note larger variability in BADE's ISE values than in VB3's. This is presumably due to a lesser degree of smoothing in BADE than in VB3.

Even though the differences in accuracy seem to be small in some cases, even a small consistent difference can be considered significant in this problem, as it has been difficult to make performance improvements in density estimation even when moving from fixed-bandwidth to variable-bandwidth methods (Terrell and Scott, 1992).

#### 3.1.2. Real examples

We tested our method on three univariate real data sets. Although not related to our intended application domain of molecular dynamics, the three data sets are widely used in the relevant statistics literature so that we can compare results easily with those from other methods:

1. **Univariate Old Faithful:** lengths, in minutes, of 107 eruptions of the Old Faithful geyser (Silverman, 1986).
2. **Suicide:** lengths, in days, of 86 treatment spells of control patients in a suicide study (Silverman, 1986).
3. **Hidalgo stamp:** paper thickness, in mm, of 485 stamps from the 1872 Hidalgo stamp issue (Izenman and Sommer, 1988). This data set is also available in the locfit package of the R software (Loader, 2013).

Results for the Old Faithful data set are shown in Figure 6, where the density estimate from our method, BADE, is compared to three others: LS (Brewer, 2000), CAKE (Ganti and Gray, 2011), and VB3 (Hazelton, 2003). We can see that the left peak matches quite well among the four methods, except that CAKE's estimate is somewhat shifted and has a wide shoulder, and LS's estimate has a lower value at this peak. As for the right peak, again CAKE's position is quite shifted to the right, and BADE's estimate shows a splitting in two submodes, which is visible just slightly in the other estimates. Finally, we observe that the other methods produce heavier tails than BADE (BADE will always produce “light,” exponential tails due to the “effective” *k* (Equation 4). This *k*_*e*_ as a function of *x* is shown in the second row of the figure, before the covariance-smoothing step.) The third row of the figure shows the standard deviation σ_*k*_(*x*) (the one-dimensional analog of *V*_*k*_(**x**)) of the set *N*_*k*_(*x*) of *k* nearest neighbors of *x*. Notice the balanced feature of the method: regions of higher *k* correspond to regions of lower σ_*k*_, and vice versa.

The density estimates for the suicide data set are shown in Figure 7. We see a very good agreement among the three methods: BADE, LS (Brewer, 2000), and CAKE (Ganti and Gray, 2011). BADE shows a small satellite mode of the main peak, where LS and CAKE exhibit a small shoulder instead. On the other hand, CAKE is significantly more sensitive than BADE and LS to the sample points around *x* = 250, *x* = 320, and *x* = 600, while BADE and LS show only a small mode at around *x* = 250 and then they taper off. In this case we can see that that the exponential decay of *k*_*e*_ is slow as *x* grows, due to the large separation of the sample points at the right end, and hence the large σ_*k*_ values in that region.

The Hidalgo stamp comparison between BADE and LS is shown in Figure 8. In contrast with the Old Faithful example, here BADE's estimate looks more smoothed than LS's, but otherwise the position and number of modes is the same for both methods. This is interesting in connection with the results of the analysis carried out by Brewer (2000), whose LS method was the only one, among the ones considered in his comparison with previous methods, that revealed exactly five modes.

### 3.2. Bivariate case

#### 3.2.1. Simulated examples

**Figure 9:**
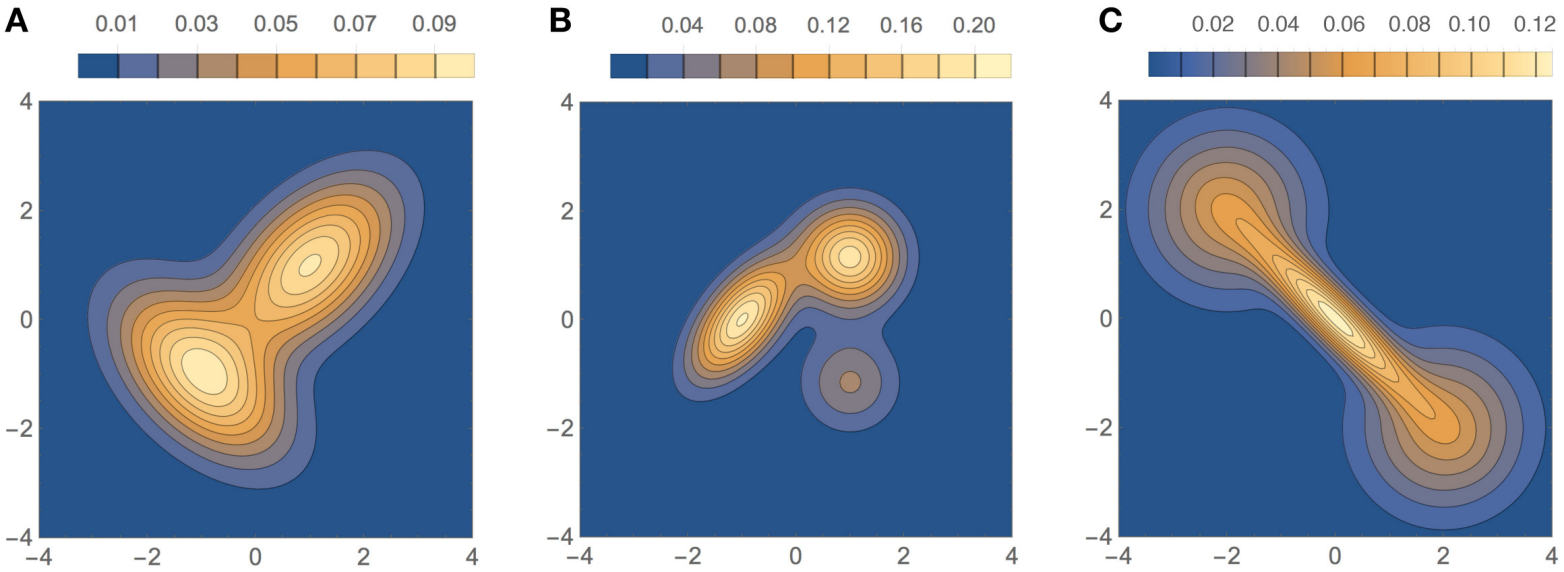
**Test densities (Gaussian mixtures) used in our bivariate simulation study**. **(A)** F2 (bimodal); **(B)** F3 (trimodal); **(C)** F4 (dumbbell unimodal).

The three bivariate simulated densities, all Gaussian mixtures, on which we tested our method are shown in Figure 9. They are densities F2, F3, and F4 used by Zougab et al. (2014), and we will refer to them by the same names:

1. **F2** (bimodal, similar to density H of Wand and Jones, 1993): $\frac{1}{2}\text{N}\left[\left(1,1\right),\Sigma_{1}\right]+\frac{1}{2}\text{N}\left[\left(-1,-1\right),\Sigma_{2}\right]$, where $\Sigma_{1}=(\begin{array}{cc} 1 & 1/2\\ 1/2 & 1 \end{array})$ and $\Sigma_{2}=(\begin{array}{cc} 1 & -1/2\\ -1/2 & 1 \end{array})$.
2. **F3** (trimodal, equal to density K of Wand and Jones, 1993): $\frac{3}{7}\text{N}\left[\left(-1,0\right),\Sigma_{1}\right]+\frac{3}{7}\text{N}\left[\left(1,2/\sqrt{3}\right),\Sigma_{2}\right]+\frac{1}{7}\text{N}\left[\left(1,-2/\sqrt{3}\right),\Sigma_{3}\right]$, where $\Sigma_{1}=\left(\begin{array}{cc} 9/25 & 63/250\\ 63/250 & 49/100 \end{array}\right)$ and $\Sigma_{2}=\Sigma_{3}=\left(\begin{array}{cc} 9/25 & 0\\ 0 & 9/25 \end{array}\right)$.
3. **F4** (“dumbbell” unimodal):$\frac{4}{11}\text{N}\left[\left(-2,2\right),\Sigma_{1}\right]+\frac{3}{11}\text{N}\left[\left(0,0\right),\Sigma_{2}\right]+\frac{4}{11}\text{N}\left[\left(2,-2\right),\Sigma_{3}\right]$, where $\Sigma_{1}=\Sigma_{3}=\left(\begin{array}{cc} 1 & 0\\ 0 & 1 \end{array}\right)$ and $\Sigma_{2}=\left(\begin{array}{cc} 0.8 & -0.72\\ -0.72 & 0.8 \end{array}\right)$.

**Figure 10:**
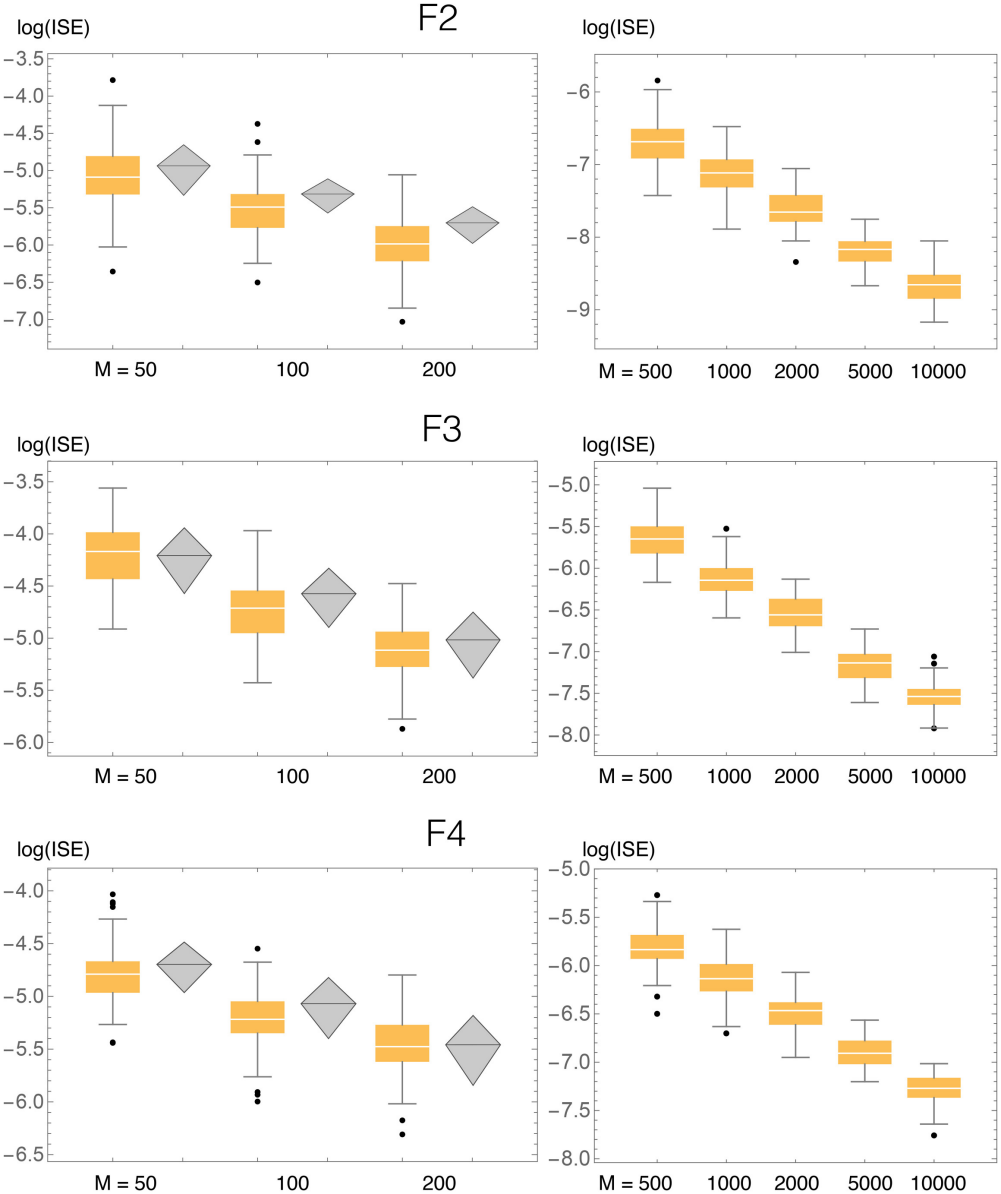
**ISE statistics for the three bivariate test densities (F2, F3, F4), comparing our method (BADE, in orange) and BABM (Zougab et al., 2014, in gray)**. Also shown are results for larger values of the sample size *M*, not considered by Zougab et al. Their results are shown by gray rhombi representing the mean plus and minus 1 standard deviation, which are the only data they reported.

Results of ISE statistics comparing our method with that of Zougab et al. (2014) are displayed in Figure 10. Zougab et al.'s results were taken directly from their paper, but since they report just the mean and standard deviation of the ISE values, we show those two parameters as rhombi, whose horizontal line corresponds to the mean, and whose top and bottom vertices correspond to ±1 standard deviation from the mean. We also considered larger sample sizes, up to 10,000. For each density and sample size, 100 simulated samples were produced. We can observe that in most cases the ISE values of our method (BADE) are lower that those of Zougab et al.'s method (BABM). The exceptions are F3 with *M* = 50 and F4 with *M* = 200, for which they are very similar.

#### 3.2.2. Real examples

We tested our method on two bivariate real data sets, and compared the results with those from other methods. Again we chose data from outside our intended application in molecular dynamics to better compare with the available statistics literature:

1. **Bivariate Old Faithful:** length of eruptions vs. interval between consecutive eruptions, for 272 observations of the Old Faithful geyser (Härdle, 1991). These data are also available on the Internet, as extra material to Wasserman's book (Wasserman, 2004).
2. **UNICEF:** under-5 mortality (number of children who died under age 5 per 1,000 live births) vs. the average life expectancy (in years) at birth, for 73 countries with Gross National Income less than US$1,000 per annum per capita. These data are available from the UNICEF and also in the ks package of the R software (Duong, 2016).

**Figure 11:**
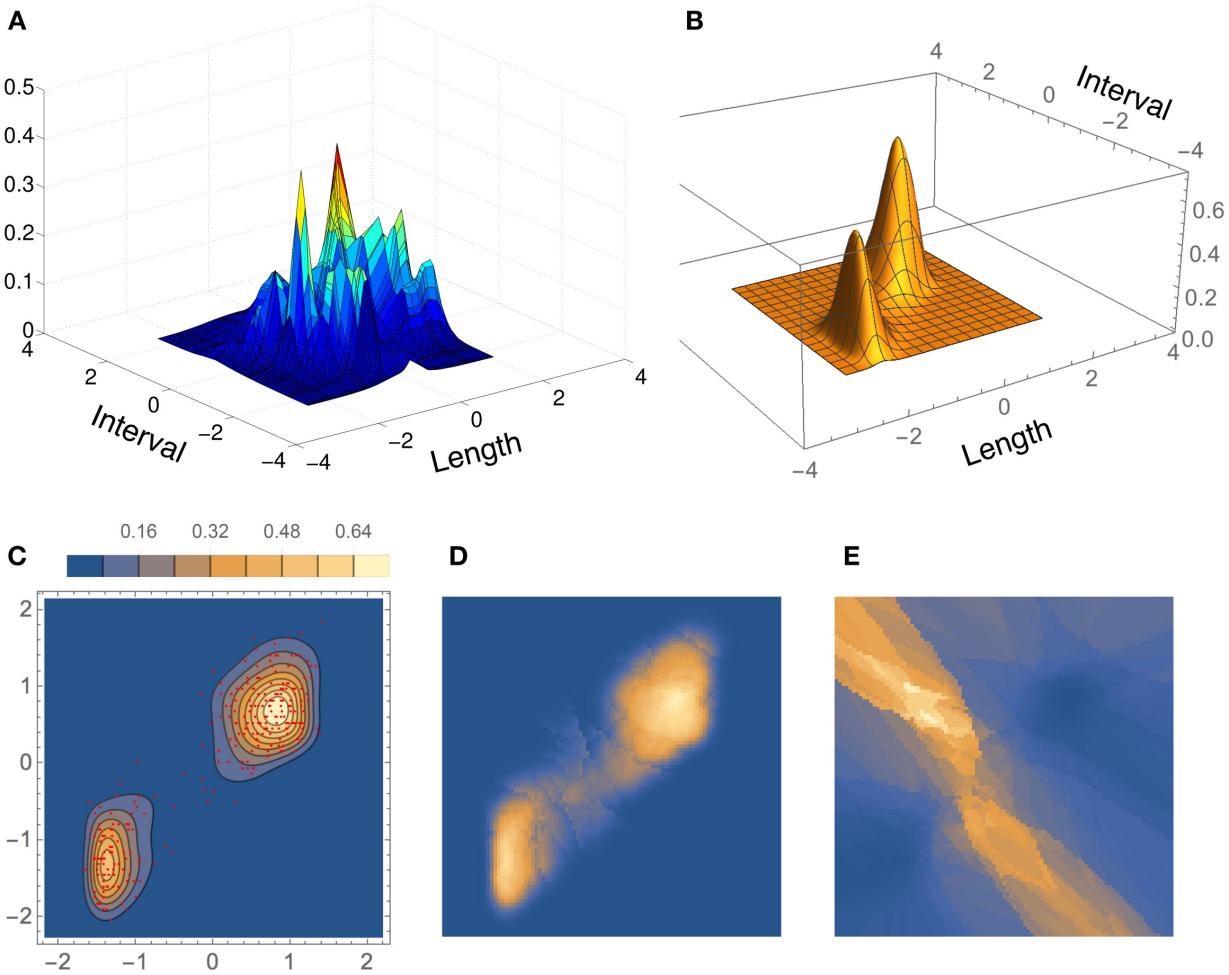
**Density estimates for the bivariate Old Faithful data set, comparing our method (BADE) with CAKE (Ganti and Gray, 2011)**. **(A)** CAKE estimate. **(B)** BADE estimate. **(C)** BADE estimate shown as a contour plot, along with the sample points. **(D)** Effective *k* values (i.e., *k*_*e*_) on a grid of size 100 × 100. **(E)** *V*_*k*_ on the same grid. Both *k*_*e*_ and *V*_*k*_ are the ones before applying the covariance smoothing step.

Results for the bivariate Old Faithful data set are shown in Figure 11, where the density estimate from our method, BADE, is compared to that from CAKE (Ganti and Gray, 2011). (In this figure, we show the scaled coordinates in order to match CAKE's plot.) Both estimates show two main peaks; however, CAKE's estimate (Figure 11A) has, in addition, many other peaks that are not present in BADE's estimate, which is a clean bimodal density (Figures 11B,C). Figures 11D,E show, respectively, the effective number of nearest neighbors, *k*_*e*_, and the area of the covariance ellipse, *V*_*k*_ (before the covariance-smoothing step). As in the univariate case, we see that regions of large *k*_*e*_ correspond to regions of small *V*_*k*_, and vice versa.

**Figure 12:**
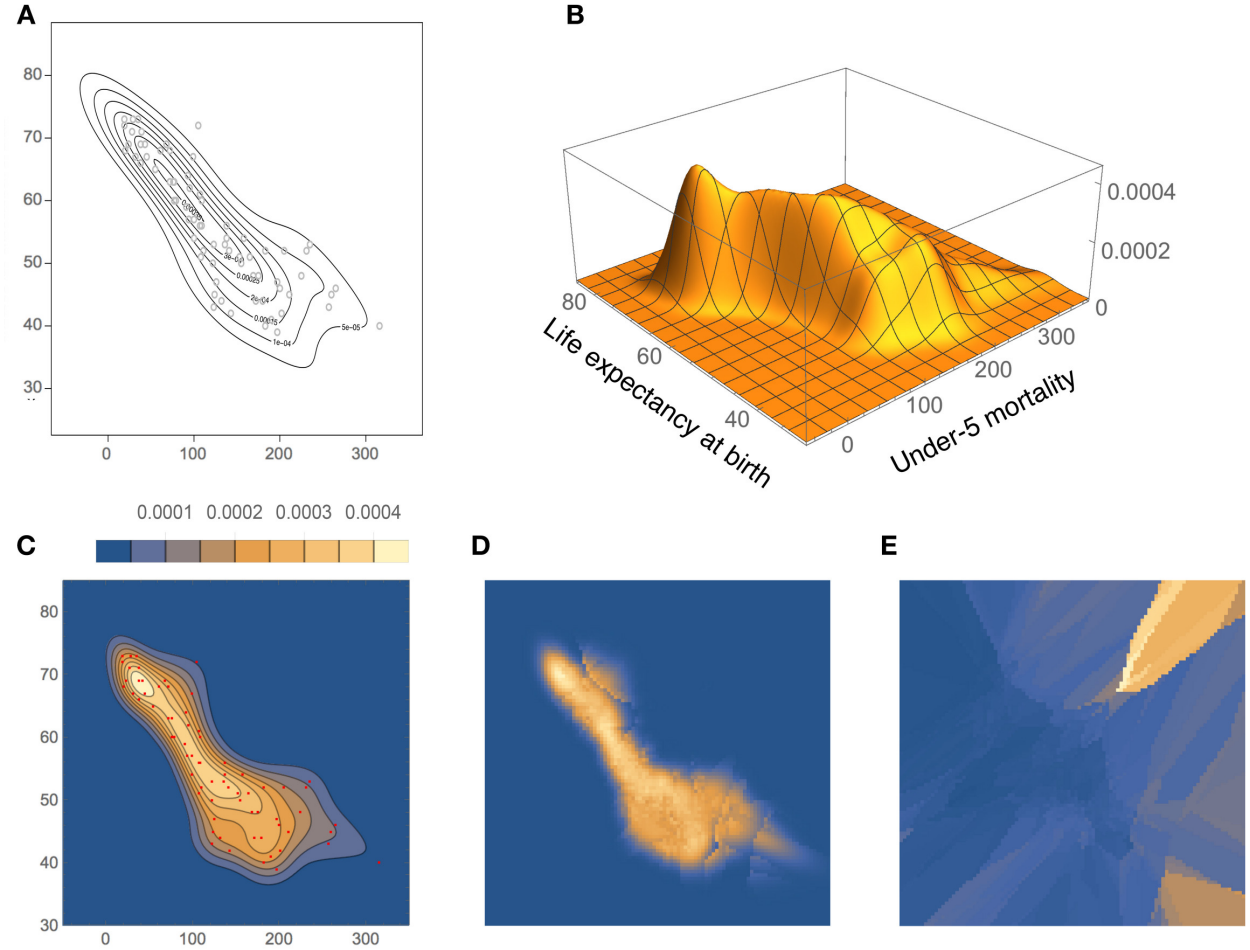
**Density estimates for the UNICEF data set, comparing our method (BADE) with BABM (Zougab et al., 2014)**. **(A)** BABM estimate. **(B)** BADE estimate. **(C)** BADE estimate shown as a contour plot, along with the sample points. **(D)** Effective *k* values (i.e., *k*_*e*_) on a grid of size 100 × 100. **(E)** *V*_*k*_ on the same grid. Both *k*_*e*_ and *V*_*k*_ are the ones before applying the covariance smoothing step.

The density estimates for the UNICEF data set, computed with our method and BABM (Zougab et al., 2014), are shown in Figure 12. Even though there is a good overall agreement between the two, BADE's estimate is apparently less smoothed than BABM's, resulting, in particular, in a shifted position of the mode toward the upper-left of the plot. In fact, BABM's estimate is virtually the same as that obtained using a fixed global bandwidth matrix (Zougab et al., 2014, Figure 3). Figures 12D,E show again the inverse relationship between *k*_*e*_ and *V*_*k*_ (before covariance smoothing).

## 4. Conclusion

We have implemented a novel adaptive density-estimation approach suitable for our statistical evaluation of membrane simulations in Wriggers et al. (2017).

Unlike most well known density estimation methods, ours is not based on kernels. Rather, it estimates the density at a given point directly, using the information about the sets of *k* nearest neighbors, finding the optimal *k* in an adaptive way, by balancing it with the size of the “covariance ellipsoid” of the set of nearest neighbors. Thus, the calculation does not involve solving costly optimization problems, and is free of data-dependent parameters. (However, in the optional smoothing step, one could vary the coefficients in Equation (11), to obtain density estimates with greater or lesser amount of smoothing).

We note that, specially in the context of fixed-bandwidth kernels, the covariance matrix could be considered a parameter which depends on the data. However, since in our approach it is not a fixed value, but rather a function of the point, we do not call it a parameter. Rather, the parameters are the coefficients in Equation (11), which are fixed (except for the optional smoothing variation) and do not depend on the data.

BADE is well suited for large data sizes. Methods that center a kernel function at each sample point become very expensive as the data size grows. Instead, BADE relies only on nearest-neighbor information, whose average required number $\overset{-}{k}\left(M\right)$ is such that $\overset{-}{k}\left(M\right)/M\to 0$ as *M* → ∞, where *M* is the sample size (Loftsgaarden and Quesenberry, 1965). Thus, the main step scales very well with data size (sublinearly in one and two dimensions, Equation 16). On the contrary, methods such as that of Zougab et al. (2014) (with which we compared ours) scale quadratically with the data size and are thus restricted to smaller data sets.

Our method is free of restrictions on the bandwidth matrices, such as diagonal or scalar. In fact, we are no longer dealing with “bandwidth” matrices, but covariance matrices of sets of nearest neighbors.

BADE has been defined for data of any dimension; however, we have worked out the constants and made tests only for dimensions 1 and 2. It is most efficient in low dimensions, due to the need to compute nearest neighbors. For this, it takes advantage of the R^*^-tree data structure (Beckmann et al., 1990), which is, to the best of our knowledge, the most efficient one for nearest-neighbor search in low dimensions. In higher dimensions the R^*^-tree data structure becomes less efficient due to the increasing relative volume of the “corners” of the hyperrectangles, and so better adapted data structures would be preferable in this case (see Hjaltason and Samet, 1999 for details).

Our method was validated, both in the univariate and the bivariate settings, by ISE analyses on some simulated densities. These analyses consisted in generating a number of simulated samples (500 for the univariate case, 100 for the bivariate case) and measuring the integrated square error (ISE) between the density estimated from each sample and the actual density function. The ISE statistics were compared with similar results from previous approaches that were among the best available. In most cases we obtained lower errors, and in the remaining few cases the performance was virtually identical.

The apparent synergy between objective (low ISE) and subjective (visual appeal) criteria in our algorithm is a curious phenomenon that has also been observed by other researchers. Farmen and Marron (1999) pointed out that “visual error appears to be quite informative about performance,” whereas Brewer (2000) stated that “subjective feeling about density estimates” produces “estimates relatively free from unnecessary minor fluctuations.” Although the earlier work provides a rationale for including subjective criteria in our work, an open research question is whether aesthetics and objective error are covariant. Farmen and Marron have attempted to quantify visual appeal (Farmen and Marron, 1999) but they found that “good performance in MISE does not guarantee visually appealing curve estimates.” In contrast, Hazelton (2003) states that “gains in ISE may understate the improvements in visual appeal,” which seems to imply at least a weak dependence. A more systematic investigation of the objective value of subjective criteria could be the subject of future work.

The optional covariance-smoothing step in BADE yields very visually appealing density estimates, as our real-data examples show, but is not strictly necessary if all that's needed is a density estimate to perform further calculations. For instance, one of the applications for which we need bivariate density estimates is the computation of Mutual Information. In this case we don't need visually appealing functions, and thus we can save significant compute time.

At this time the algorithm is implemented as a C program. It will be freely disseminated as a part of release 1.5 of our software package *TimeScapes* (Kovacs and Wriggers, 2016). The web site for our software is http://timescapes.biomachina.org.

## Author contributions

The mathematical theory of BADE was designed by JK. Experimental test data sets used in this work were prepared by CH. The larger project (including the accompanying paper) was supervised by WW. The paper was written by JK and WW.

## Funding

This work was supported in part by the Frank Batten endowment and by National Institutes of Health grant R01GM62968 (to WW).

### Conflict of interest statement

The authors declare that the research was conducted in the absence of any commercial or financial relationships that could be construed as a potential conflict of interest.

## References

[B1] Abramson I. S. (1982). On bandwidth variation in kernel estimates—A square root law. Ann. Statist. 10, 1217–1223. 10.1214/aos/1176345986

[B2] Beckmann N. Kriegel H.-P. Schneider R. Seeger B. (1990). The R^*^-tree: an efficient and robust access method for points and rectangles, in Proceedings of the 1990 ACM SIGMOD International Conference on Management of Data, SIGMOD '90 (New York, NY: ACM), 322–331. 10.1145/93597.98741

[B3] Breiman L. Meisel W. Purcell E. (1977). Variable kernel estimates of multivariate densities. Technometrics 19, 135–144. 10.1080/00401706.1977.10489521

[B4] Brewer M. J. (2000). A Bayesian model for local smoothing in kernel density estimation. Stat. Comput. 10, 299–309. 10.1023/A:1008925425102

[B5] Duong T. (2016). ks: Kernel Smoothing. R package version 1.10.3.

[B6] Farmen M. Marron J. S. (1999). An assessment of finite sample performance of adaptive methods in density estimation. Comput. Stat. Data Anal. 30, 143–168. 10.1016/S0167-9473(98)00070-X

[B7] Fredman M. L. Sedgewick R. Sleator D. D. Tarjan R. E. (1986). The pairing heap: a new form of self-adjusting heap. Algorithmica 1, 111–129. 10.1007/BF01840439

[B8] Ganti R. Gray A. (2011). CAKE: convex adaptive kernel density estimation, in Proceedings of the 14th International Conference on Artificial Intelligence and Statistics (AISTATS 2011), volume 15 of *JMLR Workshop and Conference Proceedings*, eds Gordon G. Dunson D. Dudík M. (Fort Lauderdale, FL), 498–506.

[B9] Hall P. Hu T. C. Marron J. S. (1995). Improved variable window kernel estimates of probability densities. Ann. Stat. 23, 1–10. 10.1214/aos/1176324451

[B10] Härdle W. (1991). Smoothing Techniques with Implementation in S, 1st Edn. New York, NY: Springer-Verlag.

[B11] Hazelton M. L. (2003). Variable kernel density estimation. Aust. New Zealand J. Stat. 45, 271–284. 10.1111/1467-842X.00283

[B12] Hjaltason G. R. Samet H. (1999). Distance browsing in spatial databases. ACM Trans. Database Syst. 24, 265–318. 10.1145/320248.320255

[B13] Izenman A. J. Sommer C. J. (1988). Philatelic mixtures and multimodal densities. J. Am. Stat. Assoc. 83, 941–953. 10.1080/01621459.1988.10478683

[B14] Jones M. C. Marron J. S. Sheather S. J. (1996). A brief survey of bandwidth selection for density estimation. J. Am. Stat. Assoc. 91, 401–407. 10.1080/01621459.1996.10476701

[B15] Katkovnik V. Shmulevich I. (2002). Kernel density estimation with adaptive varying window size. Patt. Recogn. Lett. 23, 1641–1648. 10.1016/S0167-8655(02)00127-7

[B16] Kovacs J. A. Wriggers W. (2016). Spatial heat maps from fast information matching of fast and slow degrees of freedom: application to molecular dynamics simulations. J. Phys. Chem. B. 120, 8473–8484. 10.1021/acs.jpcb.6b02136 27169521 PMC5545105

[B17] Liu H. Lafferty J. Wasserman L. (2007). Sparse nonparametric density estimation in high dimensions using the Rodeo, in Proceedings of the 11th International Conference on Artificial Intelligence and Statistics (AISTATS 2007), volume 2 of JMLR Workshop and Conference Proceedings, eds Meila M. Shen X. (San Juan), 283–290.

[B18] Loader C. (2013). locfit: Local Regression, Likelihood and Density Estimation. R package version 1.5-9.1.

[B19] Loftsgaarden D. O. Quesenberry C. P. (1965). A nonparametric estimate of a multivariate density function. Ann. Math. Stat. 36, 1049–1051. 10.1214/aoms/1177700079

[B20] Marron J. S. Tsybakov A. B. (1995). Visual error criteria for qualitative smoothing. J. Am. Stat. Assoc. 90, 499–507. 10.1080/01621459.1995.10476541

[B21] Marron J. S. Wand M. P. (1992). Exact mean integrated squared error. Ann. Statist. 20, 712–736. 10.1214/aos/1176348653

[B22] Sain S. R. (2002). Multivariate locally adaptive density estimation. Comput. Stat. Data Anal. 39, 165–186. 10.1016/S0167-9473(01)00053-6

[B23] Sain S. R. Scott D. W. (1996). On locally adaptive density estimation. J. Am. Stat. Assoc. 91, 1525–1534. 10.1080/01621459.1996.10476720

[B24] Sheather S. J. Jones M. C. (1991). A reliable data-based bandwidth selection method for kernel density estimation. J. R. Stat. Soc. Ser. B (Methodol.) 53, 683–690.

[B25] Shimazaki H. Shinomoto S. (2010). Kernel bandwidth optimization in spike rate estimation. J. Comput. Neurosci. 29, 171–182. 10.1007/s10827-009-0180-4 19655238 PMC2940025

[B26] Silverman B. W. (1986). Density Estimation, 1st Edn. Boca Raton, FL: Chapman & Hall/CRC.

[B27] Song L. Zhang X. Smola A. Gretton A. Schölkopf B. (2008). Tailoring density estimation via reproducing kernel moment matching, in Proceedings of the 25th International Conference on Machine Learning, ICML '08 (New York, NY: ACM), 992–999.

[B28] Terrell G. R. Scott D. W. (1992). Variable kernel density estimation. Ann. Stat. 20, 1236–1265. 10.1214/aos/1176348768

[B29] Vapnik V. Mukherjee S. (2000). Support vector method for multivariate density estimation, in Proceedings of the Conference on Neural Information Processing Systems (NIPS 1999), volume 12 of *Advances in NIPS*, eds Solla S. A. Leen T. K. Müller K. (Cambridge, MA: MIT Press), 659–665.

[B30] Wand M. P. Jones M. C. (1993). Comparison of smoothing parameterizations in bivariate kernel density estimation. J. Am. Stat. Assoc. 88, 520–528. 10.1080/01621459.1993.10476303

[B31] Wand M. P. Jones M. C. (1995). Kernel Smoothing, 1st Edn. London: Chapman and Hall.

[B32] Wasserman L. (2004). All of Statistics, 1st Edn. New York, NY: Springer.

[B33] Wriggers W. Castellani F. Kovacs J. A. Vernier P. T. (2017). Computing spatiotemporal heat maps of lipid electropore formation: a statistical approach. Front. Mol. Biosci. 4:22 10.3389/fmolb.2017.00022 PMC5404627 28487856

[B34] Wu T.-J. Chen C.-F. Chen H.-Y. (2007). A variable bandwidth selector in multivariate kernel density estimation. Stat. Prob. Lett. 77, 462–467. 10.1016/j.spl.2006.08.013

[B35] Zougab N. Adjabi S. Kokonendji C. C. (2014). Bayesian estimation of adaptive bandwidth matrices in multivariate kernel density estimation. Comput. Stat. Data Anal. 75, 28–38. 10.1016/j.csda.2014.02.002

